# Association of a placental Interleukin-6 genetic variant (rs1800796) with DNA methylation, gene expression and risk of acute chorioamnionitis

**DOI:** 10.1186/s12881-019-0768-0

**Published:** 2019-02-22

**Authors:** Chaini Konwar, Giulia F. Del Gobbo, Jefferson Terry, Wendy P. Robinson

**Affiliations:** 1 BC Children’s Hospital Research Institute, 950 W 28th Ave, Vancouver, BC V5Z 4H4 Canada; 2 Department of Medical Genetics, University of British Columbia, Vancouver, BC V6H 3N1 Canada; 3 Department of Pathology, BC Children’s Hospital, Vancouver, BC V6H 3N1 Canada

**Keywords:** Placenta, Chorioamnionitis, SNP, Interleukin-6, DNA methylation, Gene expression

## Abstract

**Background:**

Acute chorioamnionitis (aCA), inflammation of the placenta and fetal membranes, is a frequently reported lesion in preterm deliveries. Genetic variants in innate immune system genes such as Interleukin-6 (*IL6*) may contribute to the placenta’s inflammatory response, thus predisposing some pregnancies to aCA. These genetic variants may modulate molecular processes such as DNA methylation and gene expression, and in turn might affect susceptibility to aCA. Currently, there is remarkably little research on the role of fetal (placental) genetic variation in aCA. We aimed to explore the associations between genetic variants in candidate immune-system genes and susceptibility towards inflammatory responses in the placenta, which is linked to a strong inflammatory response in the newborn.

**Methods:**

DNA samples from 269 placentas (72 aCA cases, 197 non-aCA cases) were collected for this study. Samples were genotyped at 55 ancestry informative markers (AIMs) and 16 additional single nucleotide polymorphisms (SNPs) in 12 candidate innate immune system genes using the Sequenom iPLEX Gold Assay. Publicly available datasets were used to obtain DNA methylation (GSE100197, GSE74738, GSE115508, GSE44667, GSE98224) and gene expression data (GSE44711, GSE98224).

**Results:**

Differences in *IL6* placental allele frequencies were associated with aCA (rs1800796, *p* = 0.04) with the CC genotype specifically implicated (OR = 3.1; *p* = 0.02). In a subset of the placental samples (*n* = 67; chorionic villi), we showed that the *IL6* SNP (rs1800796) was associated with differential DNA methylation in five *IL6*-related CpG sites (cg01770232, cg02335517, cg07998387, cg13104385, and cg0526589), where individuals with a CC genotype showed higher DNA methylation levels than individuals carrying the GG genotype. Using two publicly available datasets, we observed that the DNA methylation levels at cg01770232 negatively correlated with *IL6* gene expression in the placenta (*r =* − 0.67, *p* < 0.004; *r =* − 0.56, *p* < 2.937e-05).

**Conclusions:**

We demonstrated that the minor C allele at the *IL6* SNP (rs1800796), which is largely limited to East Asian populations, is associated with the presence of aCA. This SNP was associated with increased DNA methylation at a nearby MEPC2 binding site, which was also associated with decreased expression of *IL6* in the placenta. Decreased expression of *IL6* may increase vulnerability to microbial infection. Additional studies are required to confirm this association in Asian populations with larger sample sizes.

**Electronic supplementary material:**

The online version of this article (10.1186/s12881-019-0768-0) contains supplementary material, which is available to authorized users.

## Background

Preterm birth (PTB) refers to all births occurring before 37 weeks of gestation, as defined by the World Health Organization [1]. PTB occurs in approximately 11% of live births worldwide, although there is substantial variability in rates on a per-country basis [2]. Children who are born preterm are at a higher risk for life-long health complications, including chronic lung disease, cerebral palsy, mental retardation, attention-deficit hyperactivity disorder, and learning disabilities [3, 4]. Clinically, PTB can be categorized into spontaneous PTB (sPTB) or medically-indicated PTB (iatrogenic) [1]. sPTB, making up the majority of PTBs, typically results from a dysregulation of inflammatory signalling pathways [5]. This often presents as acute chorioamnionitis (aCA), which is characterized by an infiltration of maternal neutrophils into the chorioamniotic membranes, typically in response to an ascending microbial infection from the genital tract. This acute placental inflammation can also be triggered by non-microbial “danger signals” including cellular stress and/or cell death [6, 7].

Genetic susceptibility for aCA can be hypothesized based on: i) high heritability estimates of PTB (15–30%) [8, 9], ii) evidence supporting familial segregation of PTB [10, 11], (iii) association of placental histopathological inflammatory lesions with recurrent PTB [12, 13], and (iv) ethnic disparities in PTB [14–16] and chorioamnionitis rates [17]. Inherited differences in immune system genes also influence susceptibility to microbial infection [18–21], which is a well-known cause of aCA. In addition, a strong genetic predisposition underlies many infectious and inflammatory diseases, particularly in early childhood [22–24]; this may also hold true for in utero susceptibility for aCA.

Studies investigating candidate genes have reported that maternal and fetal genetic variation in Toll-like receptors (*TLRs*) is associated with sPTB [25–27]. Allelic variation in *TLR* genes has been shown to modulate immune responses during parturition, and thus confer an altered risk of preterm delivery [28]. Genetic variants in cytokine genes such as Interleukin-6 (*IL6*) have also been associated with intrauterine infection and/or inflammation in sPTB [29–31]. Furthermore, elevated concentration of *IL6* in maternal serum, cervical secretions and amniotic fluid are associated with sPTB [32–35]. Recently, a genome-wide association study investigating > 40,000 women of European ancestry identified several genetic variants associated with sPTB [36]. Although variants in *EBF1, EEFSEC,* and *AGTR2* genes were replicated in an independent cohort of > 8000 women, none of the identified genes had been previously identified in sPTB or known to have a direct role in inflammatory mechanisms [36]. While these studies have provided some insight on genetic variation linked to sPTB, rarely are the same loci reported with sPTB risk. sPTB is heterogeneous in etiology [37], thus inconsistent phenotyping of sPTB cases and differences in population structure may explain the discrepancies across these studies.

Genetic variants within coding regions may directly affect protein function, while those in regulatory regions may affect molecular processes such as DNA methylation [38–40] that are involved in regulating gene expression [41, 42]. Alternatively, genetic variants can alter the binding site of transcription factors and affect gene expression, which then influences DNA methylation levels, suggesting DNA methylation as a consequence of gene regulation [43]. Irrespective of the underlying mechanism, these effects can in turn affect susceptibility to inflammatory diseases. For example in rheumatoid arthritis, DNA methylation at an *IL6*-related CpG was altered in affected patients, and a negative relationship between DNA methylation and *IL6* mRNA levels was observed, suggesting a DNA methylation-dependent regulation of *IL6* transcription [44]. While increased serum levels of *IL6* have been previously reported in aCA [45–47], these studies did not take into account the genotype at the *IL6* locus and/or the DNA methylation status of the *IL6*-related CpGs. Furthermore, the maternal genotype is often investigated although the placental genotype may be more relevant in terms of mediating pregnancy-related inflammation. Elucidating these complex relationships between genotype, DNA methylation and gene expression is important to improve our understanding of the genetic regulation of placental inflammation.

In this study, we investigated the association between 16 candidate SNPs in 12 innate immune system genes and the presence of aCA. These SNPs were chosen based on published reports of an association with chorioamnionitis [48–50], placental inflammation [51, 52] or neonatal sepsis/infection [53–55]. We validated these associations in a population of 269 placentas, of which 72 were affected with aCA and 197 were unaffected (non-aCA). Further, we investigated whether aCA-associated SNPs showed also a correlation with altered DNA methylation of the associated gene, and determined whether DNA methylation levels correlated with gene expression.

## Methods

### Study cohort

Ethics approval was obtained from the University of British Columbia Children’s & Women’s Research Ethics Board (H04–70488). The study cohort is based on an ongoing collection of samples for our Epigenetics in Pregnancy study (EPIC), and overlaps with samples described in previous publications related to placental DNA methylation [56–58]. Most cases were obtained in a deidentified manner with pathology and birth information available, but limited information on maternal health or demographics.

Placentas were collected from pregnancies delivered at the Children’s & Women’s Health Centre, Vancouver, Canada. Placentas from 72 aCA cases were selected based on a diagnosis of aCA determined by pathological examination of the placenta and associated membranes using consensus histological criteria [59]. Another set of 197 non-aCA cases were identified from this same collection of placentas. These consisted of 73 PTBs with no evidence of aCA and/or placental inflammation including cases of spontaneous premature preterm rupture of the membranes, placental abruption, fetal vascular malperfusion, acute hypoxic ischemic event, and preterm labor), in addition to 124 term (> 37 weeks gestation) cases from healthy, uncomplicated pregnancies. Criteria for exclusion were fetal and/or placental chromosomal abnormalities, fetal malformations, congenital abnormalities, intrauterine growth restriction [60], preeclampsia (PE) [61], and hypertension.

Demographic characteristics of the study cohort is presented in Table 1. Of the variables investigated gestational age (GA) at delivery was significantly different between the aCA cases and non-aCA cases in our study cohort as aCA cases are associated with PTB. Although male fetuses are reported to be at an increased risk of adverse pregnancy outcomes including chronic inflammation, neonatal sepsis, and stillbirth; in our study, fetal sex was not significantly different between aCA and non-aCA groups.

**Table 1:** Identification of variables confounded with acute chorioamnionitis status (pathology)

	aCA	Non-aCA	*p*-value*
N	72	197	
Maternal age (yrs); range (mean)	19.6–44.0 (32.0)	17.0–43.5 (33.0)	ns
GA at delivery (wks); range (mean)	20.0–40.0 (28.0)	19.4–41.9 (36.0)	4.32e-15
Birth weight (SD); range (mean)	− 3.10 - 3.05 (− 0.14)	−3.13 - 3.23 (0.04)	ns
Sex; M/F	38/34	103/94	ns

*p-values are calculated by comparison of aCA cases to non-aCA cases using Wilcoxon-Mann-Whitney rank sum test for continuous variables, Fisher’s exact test for fetal sex. ns = *p* > 0.05

Further, the aCA cases and the non-aCA cases were sampled from a single urban population (Vancouver) and delivered at a single centre, BC Children’s & Women’s Health Centre which is located in a high socio-economic status neighborhood. Of the documented observations for maternal smoking status (80/269), almost all (79/80) identified themselves as non-smokers. Additionally, the most reproducible finding between maternal smoking and altered DNA methylation is observed at sites linked with AHRR and CYP1A1. We therefore tested for differences at these sites and did not observe altered DNA methylation associated with our pathology at these sites in our study cohort (data not shown).

Chorionic villus samples were obtained from the fetal side of the placenta at multiple distinct sites (cotyledons). Samples were thoroughly washed with phosphate-buffered saline to avoid maternal blood and amniotic fluid contamination. Placental DNA was extracted by a standard salting out procedure, modified from Miller et al. [62]. A NanoDrop 1000 spectrophotometer (ThermoScientific, USA) was used to assess DNA purity and concentration.

### Candidate single nucleotide polymorphism (SNP) selection

SNPs in 12 innate immune system genes were chosen based on published findings of an association with any of chorioamnionitis [48–50], placental inflammation [51, 52], or neonatal sepsis/infection [53–55]. The estimates of the minor allele frequency for the 16 SNPs varied between > 1–48% in the general population based on 1000 Genomes Project Phase III records [63]. Table 2 provides a detailed description of the 16 candidate SNPs investigated in the study.

**Table 2:** Information for 16 candidate SNPs in innate immune system genes

Genes	Gene name	Chromosome	SNPs	Genomic location
*MBL2*	Mannose binding lectin 2	10	rs1800450	Exon
*TLR2*	Toll-like receptor 2	4	rs3804099	Exon
*TLR4*	Toll-like receptor 4	9	rs1554973	3′ UTR
			rs4986790	Exon
			rs2149356	Intron
*TLR5*	Toll-like receptor 5	1	rs5744105	Intron
*TLR9*	Toll-like receptor 9	3	rs352140	Exon
*CD14*	Cluster of differentiation 14	5	rs2569190	5′UTR
*IL6R*	Interleukin-6 receptor	1	rs2228144	Exon
*IL6*	Interleukin-6	7	rs1800795	Promoter
			rs1800796	Promoter
*IL1B*	Interleukin-1 beta	2	rs1143643	Intron
*IL10*	Interleukin-10	1	rs1800896	Promoter
			rs2222202	Intron
*IL8*	Interleukin-8	4	rs4073	Promoter
*MMP-16*	Matrix metalloproteinase-16	8	rs2664349	Intron

*This information is obtained from dbSNP: database from short genetic variations (https://www.ncbi.nlm.nih.gov/snp/)

Genetic association studies are prone to population stratification if case and control groups are not matched by ancestry. Because ancestry was largely unknown in the study cohort, we also selected 55 ancestry informative marker (AIM) SNPs to genotype in our population as previously described [56]. These AIM SNPs were designed to differentiate between African, European, East Asian, and South Asian ancestries [64–66].

### Genotyping

All samples were genotyped using the Sequenom iPlex Gold platform at the Génome Québec Innovation Centre (Montréal, Canada). Primary quality control of the genotype data comprised of the following steps i) removal of samples with call rate < 90% (*n* = 1), and ii) removal of SNPs with call rate < 90% (*n* = 5; 4 AIM SNPs (rs11779571, rs2304925, rs5030240, rs917118) and 1 aCA candidate SNP (rs4986790). After primary quality control, each gene involving multiple SNPs was investigated for linkage disequilibrium (LD) to determine if the SNPs were independent of one another. Strong evidence for LD was observed for two SNP pairs (rs1800795–rs1800796 in *IL6* and rs1800896–rs2222202 in *IL10*) with D’ = 0.99. Haplotype analysis was performed for these two SNP pairs to investigate whether carriers of a specific haplotype had increased susceptibility for aCA. The 15 SNPs were also tested for Hardy-Weinberg Equilibrium (HWE) to detect genotyping error and/or population stratification.

### Inferring ancestry of the study population

After initial quality control, 50 AIM SNPs were used to infer ancestry in 269 placental villus DNA samples. Ancestry was described using the top 3 coordinates derived from a multidimensional scaling (MDS) analysis of AIM SNP genotypes of the placental villus samples and *n* = 21,571,000 Genomes Project samples (*n* = 661 African, *n* = 504 East Asian, n = 504 European, *n* = 489 South Asian) used as ancestry reference populations, as described in Del Gobbo et al. (2018) [56]. Using this method, ancestry is assessed as a continuous measure, which is relevant in our genetically heterogeneous study population. Distributions of the three ancestry MDS coordinates were compared between aCA and non-aCA groups to identify any population stratification by ancestry. To select homogenous ancestry groups from our placental chorionic villus samples, an unsupervised clustering method, *k-*means clustering, with *k* = 3, was used to cluster samples into three groups of common ancestry.

### Publicly available datasets

To investigate whether candidate SNPs were associated with altered DNA methylation, we used our previously obtained DNA methylation microarray data that was available for a subset of placental (chorionic villus) samples (*n* = 67). Some of these (GSE100197 and GSE74738; *n* = 25) were assessed on the Illumina Infinium HumanMethylation450 BeadChip (450 K array; 485,512 CpG sites) [67] while the remaining samples (GSE115508; *n* = 42) were assessed on the Illumina Infinium HumanMethylationEPIC BeadChip (850 k array; 866,895 CpG sites) [68]. Probes for 453,093 CpG sites were present on both arrays. Probe filtering was performed as described in Konwar et al. 2018 [58]. To account for type I- type II probe bias on the DNA methylation arrays, normalization was performed using ‘preprocessFunnorm’ function in the R *minfi* package [69]. Principal component analysis (PCA) was used to detect sources of variability in the DNA methylation dataset. Known technical variation associated with array type was corrected with the function “ComBat” in R *sva* package [70]. DNA methylation values were reported as β values ranging from 0 to 1 (0 = no methylation, 1 = fully methylated) and were used for biological interpretation. However, log-transformed β values, M values, were used for statistical analyses as they are less heteroscedastic [71].

Further, we took advantage of publicly available datasets to determine whether DNA methylation levels correlated with gene expression at the associated gene. Our group has previously published gene expression data (GSE44711) [72] on a set of 16 chorionic villus samples that were also run on the 450 K array to measure DNA methylation (GSE44667) [72]. Another set of 48 matched chorionic villus samples were also utilized to evaluate correlation between gene expression and DNA methylation levels (GSE98224) [73]. DNA methylation data was already normalized and corrected for batch effects. Log2-transformed expression values were used for statistical analysis.

### Statistical analysis

All statistical analyses were performed using R version 3.4.1. *p*-values for Table 1 were calculated by Wilcoxon-Mann-Whitney rank sum test for continuous variables and Fisher’s exact test for categorical variables. Kolmogorov-Smirnov (KS) test was used to assess the differences in distribution of ancestry MDS coordinate values between aCA cases and non-aCA cases. Deviation from HWE in controls was assessed using an exact test for HWE. Statistical tests for differences in allele frequencies between aCA cases and non-aCA cases were conducted with Fisher’s Exact tests. Haplotype analysis was performed using SNPStats (https://www.snpstats.net/start.htm) [74]. Comparison of DNA methylation levels between genotype groups was carried out using the non-parametric Kruskal-Wallis test. Spearman’s correlations were conducted to determine whether DNA methylation levels correlated with gene expression at the associated gene. Power was calculated using the Online Sample Size estimator, OSSE (http://osse.bii.a-star.edu.sg/index.php).

## Results

### Characterization of population stratification in study population

**Fig. 1:**
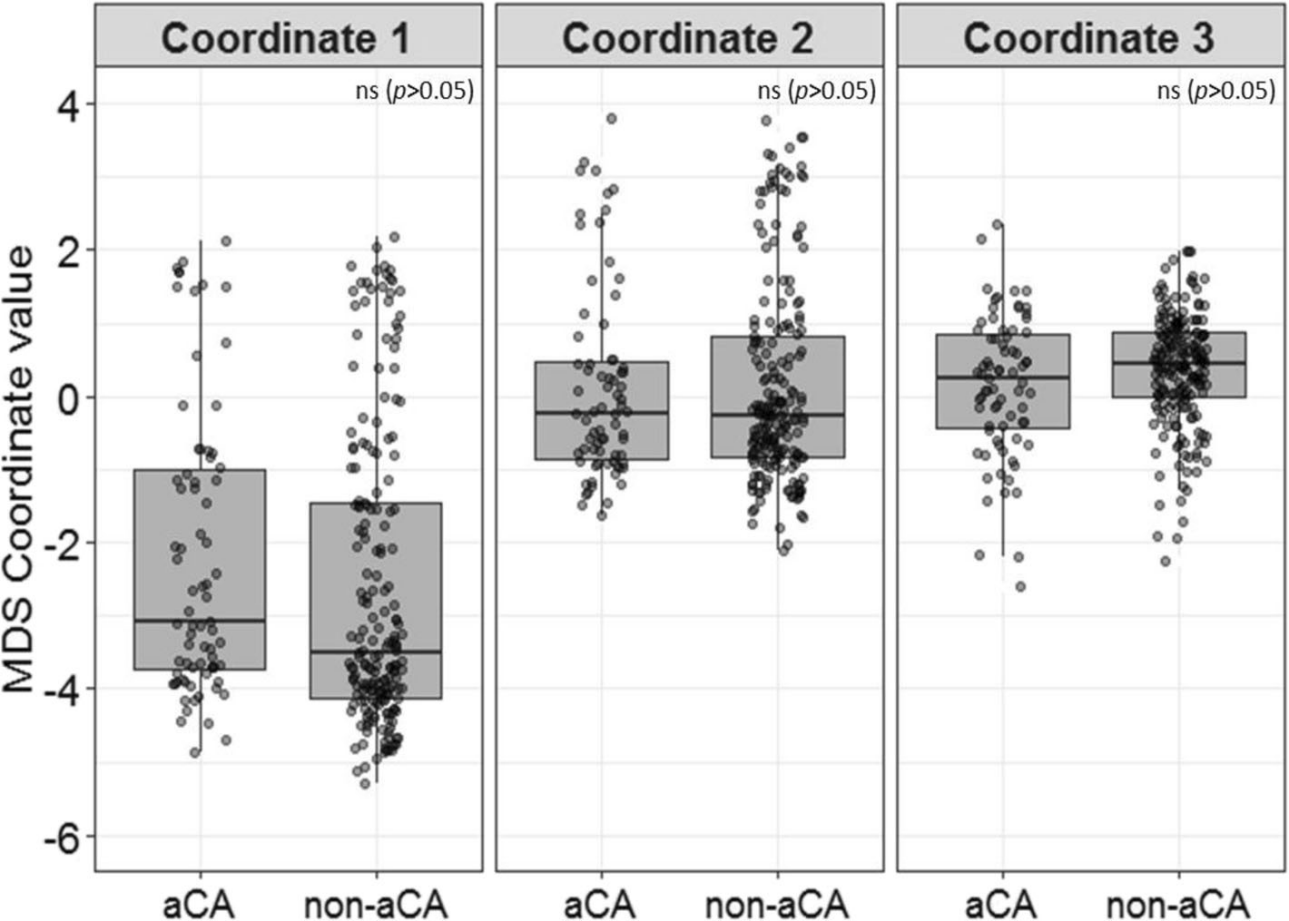
Distribution of ancestry MDS coordinates in the study cohort. The three ancestry MDS coordinates were not significantly different between the aCA cases and non-aCA cases in the study cohort, suggesting pathology was not confounded by ancestry in our study population. *p*-values were calculated by Kolmogorov-Smirnov test

It is important to determine whether pathology showed evidence of confounding with ancestry, as frequencies of genetic variants and the incidence of chorioamnionitis often vary between different ancestries [17]. There were no significant differences in the distribution of the three ancestry MDS coordinates between the 72 aCA cases and 197 non-aCA cases (Bonferroni-corrected *p >* 0.05, Fig. 1). However, we observed that ancestry MDS coordinate 1, which largely separates European and East Asians, shows the greatest variability while ancestry MDS coordinate 3, which largely separates the Europeans and South East Asians, shows the least variability. Genotype frequencies did not conform to HWE expectations in three SNPs (rs1800795, rs1800796, and rs1554973, *p* < 0.01). Because rs1800796, rs1800795 and rs1554973 deviated from HWE, we next investigated whether the frequencies of these genetic variants were associated with ancestry. Significant differences in the distribution of the two ancestry MDS coordinates between the SNP genotypes was observed (Additional file 1: Figure S1), confirming that deviation from HWE at these loci was due to our heterogeneous study cohort. Additionally, both rs1800795 and rs1800796 are in LD and do not conform to HWE, thus it is unlikely that these results are due to an artefact of genotyping.

### Association of candidate immune SNP allele frequencies with acute chorioamnionitis

To investigate whether placental (fetal) genetic variation may lead to an increased susceptibility to developing aCA, we genotyped 16 SNPs within 12 innate immune system genes among our study cohort samples (72 aCA, 197 non-aCA). We limited our analysis to SNPs previously implicated in aCA or related phenomenon. Given our small sample size, we estimated that our study had 50% power to detect differences in allele frequencies associated with aCA, at a *p*-value < 0.05, MAF > 1%. Despite this limitation, we found that the minor C allele at rs1800796 in *IL6* was associated with aCA (Fisher’s exact test, *p* = 0.044). (Additional file 2: Table S1). To explore this further we looked at the genotype distributions for rs1800796 and found that the CC genotype was associated with increased risk for aCA (Fisher’s exact test, *p* = 0.02, OR = 3.1).

**Fig. 2:**
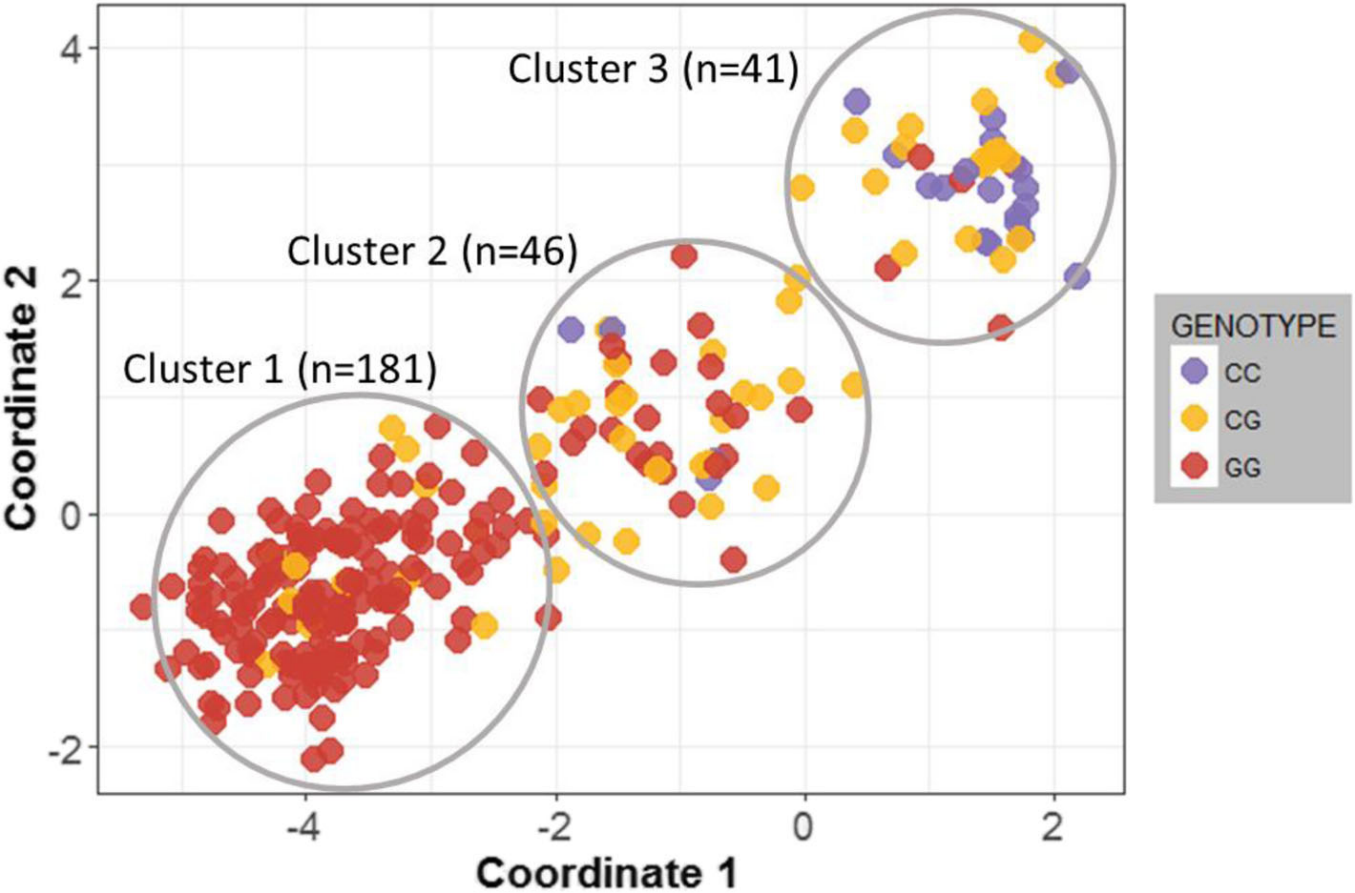
rs1800796 genotype is associated with ancestry. Based on the ancestry MDS coordinate values, three ancestry clusters were identified by *k*-means clustering. Of the 22 cases with CC genotype, 11 (50%) were associated with aCA, while 22% (60/246) of the CG/GG genotypes were linked to aCA. Samples are colored by *IL6* rs1800796 genotype

The *IL6* rs1800796 SNP is known to be strongly correlated with ancestry. Based on 1000 Genomes Project data, the C allele is common in individuals of East Asian (70–80%), and rare in individuals of European ancestry (3–5%) (https://www.ncbi.nlm.nih.gov/variation/tools/1000genomes/). We also found that the genotype frequencies of this SNP varied between different ancestries in our study population (Additional file 1: Figure S1). We thus sought to explore the association of rs1800796 with aCA within more genetically homogeneous subpopulations. *K*-means clustering grouped samples into three ancestry clusters in our study cohort. The CC genotype was absent in cluster 1, which was predominantly of European ancestry (Fig. 2 and Additional file 2: Table S2). Allele frequencies of rs1800796 were significantly associated with aCA status only in cluster 3 samples (*n* = 41) that were largely of East Asian ancestry (Fisher’s exact test, *p* = 0.04). Specifically, 8 of the 12 (67%) cases of aCA in cluster 3 had the “CC” genotype as compared to 10 of 29 (34%) non-CA cases. In our study cohort, rs1800796 was in nearly complete LD with rs1800795 (D’ = 0.99); however, unlike rs1800796, rs1800795 was polymorphic in individuals of European ancestry (Additional file 2: Table S2) and uninformative in the East Asian cluster 3 samples as the GG genotype was completely absent. Although allele frequencies in rs1800795 alone were not associated with aCA, there was an increased risk of aCA in carriers of the C-C haplotype (*p* = 0.02).

### Association of *IL6* rs1800796 genotype with DNA methylation in *IL6*

**Fig. 3:**
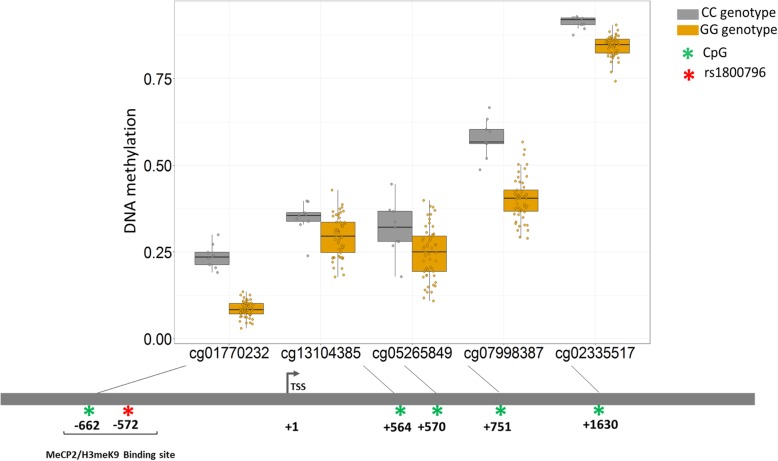
Differentially methylated *IL6* CpGs in chorionic villi associated with *IL6* rs1800786 genotype. Unadjusted DNA methylation (β) values are plotted on the y-axis w.r.t the CpGs on the x-axis. Carriers of CC genotype showed increased DNA methylation levels compared to carriers of GG genotype, suggesting DNA methylation levels at these CpG sites are influenced by *IL6* genotype. Position of the CpGs are indicated below the boxplots relative to the transcription start site (TSS) (UCSC GRCh37/hg19)

A few studies have investigated the relationship between genetic variants in *IL6* and DNA methylation of the CpGs in the promoter region of *IL6* as a mechanism by which genetic variants may modulate disease risk [75–77]. As these studies had been done in blood, we sought to examine whether the *IL6* SNP rs1800796 was associated with differential DNA methylation in *IL6*-related CpG sites (*n* = 8 CpGs) in a subset chorionic villus samples for which DNA methylation microarray data were available (*n* = 67). Modest (r > 0.5) to strong (r > 0.7) correlations were observed between β values across most of the CpG sites (Additional file 1: Figure S2). DNA methylation was significantly associated with rs1800796 genotype at cg01770232 (upstream enhancer, *p* < 1.88e-06), cg02335517 (intronic, *p* < 6.007e-06), cg07998387 (intronic, *p* < 4.968e-06), cg13104385 (intronic, *p* < 0.0003); and cg0526589 (intronic, *p* < 0.002), whereby homozygous C samples showed significant hypermethylation compared to homozygous G samples (Fig. 3). These sites have been previously identified as linked to methylation quantitative trait loci (mQTL) in blood [78], meaning CpGs where individual genotypes may result in different DNA methylation patterns [41]. The CG genotype was present in only four samples, therefore we did not include them in statistical analysis, though as expected, the heterozygotes showed intermediate DNA methylation levels (Additional file 1: Figure S3). Further, altered DNA methylation at cg01770232, cg7998387, and cg02335517 were also associated with aCA (*p* < 0.05) (Additional file 1: Figure S4).

### Correlation of DNA methylation and gene expression for *IL6*

**Fig. 4:**
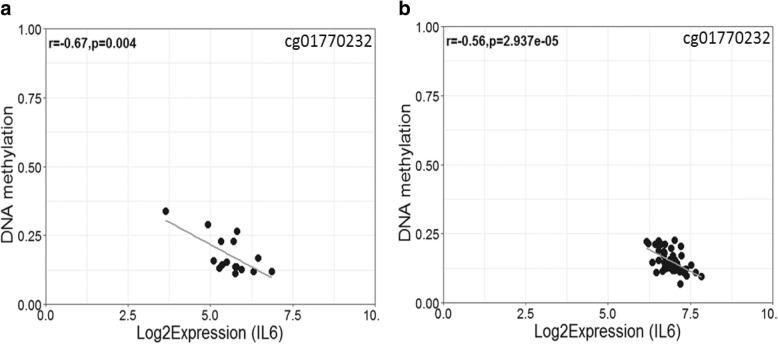
Placental DNA methylation at cg01770232 is associated with *IL6* gene expression. DNA methylation at the *IL6* CpG site was negatively correlated with *IL6* log2 transformed gene expression in chorionic villi from **a**) GSE44711; GSE44667 (*r =* − 0.67, *p* = 0.004), and **b**) GSE98224 (*r =* − 0.56, *p* = 2.937e-05)

To evaluate whether DNA methylation is associated with altered gene transcription activity, we investigated whether DNA methylation levels of the *IL6* CpGs correlated with gene expression at the *IL6* locus in chorionic villi. Using two independent publicly available datasets, we observed that the DNA methylation level at cg01770232 was negatively correlated with *IL6* expression (GSE44711; GSE44667: *r =* − 0.67, *p* < 0.004; GSE98224: *r =* − 0.56, *p* < 2.937e-05) (Fig. 4). Similar trends were observed for cg02335517, cg07998387, and cg13104385 (Additional file 1: Figure S5).

### Association of *IL6* expression and DNA methylation with GA, fetal sex or preeclampsia status

Using the GSE98224 dataset, we observed that *IL6* expression in chorionic villus samples was not associated with GA or fetal sex (Additional file 1: Figure S6). Although previous studies found altered *IL6* expression in placentas from preeclamptic pregnancies, there was no significant difference in *IL6* expression between PE and non-PE groups in this dataset (Additional file 1: Figure S6). Using the same dataset, we also did not observe an association between DNA methylation at the *IL6*-related CpGs and GA, fetal sex or PE status.

## Discussion

In this study, we sought to validate associations of SNPs in innate immune system genes with aCA status in the placenta. As the placenta and fetus are genetically identical, risk-conferring genetic variants in innate immune system genes may impact inflammation-response pathways in the placenta and fetus similarly. Additionally, the placenta employs a number of mechanisms to protect the developing fetus from inflammation and/or infection [79, 80]. Few studies have reported an association between genetic variants in inflammatory genes, alterations in immune function and risk for aCA. The majority of these studies suggest a genetic predisposition of the mother to aCA [48, 49], but the contribution of fetal (placental) genetic variants are significantly understudied.

In our study sample, we were able to confirm that the C allele in the *IL6* SNP rs1800796 was associated with aCA status (*p* = 0.04). This allele was linked to increased DNA methylation, at both an upstream regulatory region and within the gene body, and associated with decreased expression of *IL6*.

Interleukin-6 is a pleiotropic cytokine with a wide range of biological functions [81]. Primarily, IL6 facilitates neutrophil recruitment and their subsequent clearance from the sites of inflammation [82]. In addition to eliciting innate immune responses, IL6 also regulates adaptive immunity by influencing proliferation and maturation of T cells and B cells [81]. As such, increased IL6 has been shown to be protective against bacterial infection [83]. Therefore, decreased expression observed in the placenta in association with rs1800796 may lead to increased risk for inflammation and aCA.

The *IL6* SNPs rs1800796 and rs1800795 were previously associated with increased incidence of aCA and development of sepsis in children in a study undertaken in Finland [30]. In the cases of rs1800796, the heterozygous “CG” genotype was found to be associated with sepsis, and in fact the “CC” genotype was absent from their study population. These same genetic variants have also been investigated in association with other inflammatory disorders including chronic periodontitis, systemic onset juvenile chronic arthritis, and distal interphalangeal osteoarthritis [84–87]; however, results from these studies are conflicting. Inconsistencies across these studies may be explained by ancestry differences in the study populations as the CC genotype at rs1800796 is common in East Asians and rare in individuals of European ancestry [85, 88, 89]. Though a small sample, we also found no CC genotypes in the placentas of individuals of European ancestry and the C allele was more common in individuals of East Asian ancestry (Fig. 2). Further, we found that the allele frequencies of rs1800796 were associated with aCA status only in individuals of East Asian ancestry, however there was poor power to detect an effect in Europeans as the CC genotype was lacking and heterozygotes are expected to have less of an effect. Similarly, variants at the *PGR* locus, specifically the *PGR* SNP rs11224580, that significantly modulates PGR expression in the ovary has been shown to be common in East Asians compared to individuals of European and African ancestry [90], but in this case the Asian-specific variant is linked with decreased incidence of early sPTBs [90]. Because polymorphisms such as rs1800796 (*IL6*) and rs11224580 (*PGR*) exhibit extreme population-specific allelic variation, these genomic loci are likely to have undergone positive selection that is specific to Asian populations [90, 91].

In addition to genetic variation, circulating levels of IL6 in the serum and amniotic fluid may influence the risk for aCA. Although placental trophoblast cells have the capacity to synthesize IL6 [92, 93], the source of elevated IL6 plasma levels observed in pregnancy complications such as PE is primarily attributed to maternal leukocytes and/or endothelial cells [94]. Elevated IL6 levels in the mother may occur as part of a pro-inflammatory response to infection. In contrast, we found that the placental genotype (rs1800796) associated with aCA is linked with decreased *IL6* expression in the placenta*.* As *IL6* mediates a protective immune response against microbial infections [83, 95–98], reduced *IL6* expression observed in the placenta may predispose an individual to infection, by preventing an appropriate innate immune response to microbes.

Although the role of *IL6* in modulating innate immune response has been well-elucidated, molecular mechanisms such as DNA methylation underlying the genetic regulation of *IL6* transcription have rarely been investigated [99]. To our knowledge, this is the first study to show the role of *IL6* genetic variants in modulating DNA methylation patterns in aCA-affected placentas. We identified that carriers of the rs1800796 C allele had increased placental DNA methylation levels at multiple IL6*-*related CpGs, most significantly at cg01770232, which is located at an upstream enhancer, compared to individuals with *IL6* G allele. Although this CpG has been described as linked to an mQTL in blood, we showed that this relationship also exists in the placenta. Further, we observed that aCA cases were more methylated than the non-aCA cases at cg01770232, cg07998387, and cg02335517 (Additional file 1: Figure S4).

The DNA methylation patterns in the placenta may imply a primary phenomenon where increased DNA methylation at cg01770232 is associated with an increased risk of developing aCA in individuals carrying the *IL6* C allele. Alternatively, some of these subtle DNA methylation changes could be secondary to the disease itself. Although we were not able to measure circulating levels of IL6, using publicly available matched placental DNA methylation and gene expression data, we observed that DNA methylation levels at cg01770232 negatively correlated with *IL6* gene expression. Dandrea et al. (2009) [99] demonstrated that *IL6* repression in pancreatic adenocarcinoma cell lines is facilitated by binding of methyl-CpG-binding protein (MeCP2) and H3meK9 to the methylated CpGs spanning from positions − 666 to − 426 relative from the transcription start site of *IL6*. Interestingly, rs1800796 and cg01770232 is located at position 572 and 662 respectively, and it is therefore possible that rs1800796 alters the binding of MeCP2 and H3meK9 to cg01770232, thereby affecting IL6 expression and DNA methylation, though this has not been tested in placental tissue. Further, the *IL6* upstream region contains several (A/T)_> 4_ motifs adjacent to the methylated CpGs including cg01770232, shown to mediate high-affinity MeCP2 binding [100]. Understanding these mechanisms will provide insights into how genetic variation in *IL6* may contribute towards disease pathogenesis in aCA.

Overall, the present study has limitations. Our sample size was relatively small, especially among the genetically homogenous subpopulations; therefore the findings of this study should be evaluated in larger subpopulations of different ancestries. In particular, the lack of an association between the other genetic variants we investigated and aCA might be a result of our small sample size. Although we utilized publicly available datasets to investigate functional consequences of the *IL6* rs1800796 polymorphism, and confirmed that DNA methylation changes correlated with changes in *IL6* gene expression, we could not examine whether IL6 protein levels in maternal blood would reflect placental DNA methylation and expression. Further, potential confounding factors for our study, such as socio-economic status, maternal smoking status, maternal alcohol use, and PPROM status, were not documented for all the cases in our study cohort and thus not accounted for in statistical analyses. Finally, our results only highlight the biological significance of placental (fetal) genetic variants in aCA, but this does not exclude the role of maternal genetic factors in altering disease risk to aCA, given that maternal genetic effects have been shown as important contributors to PTB [10].

## Conclusion

Our findings suggest that a placental (fetal) CC genotype at the *IL6* SNP (rs1800796), which is largely limited to individuals of East Asian ancestry, is associated with aCA. Placentas with the CC genotype exhibited increased DNA methylation at multiple CpG sites upstream and within *IL6*, as compared to those of GG genotype. This increased DNA methylation was associated with lower expression of *IL6* and with aCA status. While overexpression of *IL6* is associated with various inflammatory conditions, this can be a consequence of infection; whereas innate *IL6* deficiency can lead to impaired immunity against microbial infection. Taken together, we conclude that *IL6* genetic polymorphisms may influence susceptibility to aCA by affecting the risk of acute infection. Larger samples sizes are needed to confirm these findings.

## Additional files

Additional file 1: **Figure S1.** Shows the association of SNP genotypes (rs1800795, rs1800796, and rs1554973) with ancestry in study cohort. **Figure S2.** Shows the correlation of β values across eight *IL6*-related CpGs. **Figure S3.** Shows differential methylation of *IL6-*related CpGs based on *IL6* genotype status (rs1800796). **Figure S4.** Shows altered DNA methylation at *IL6-*related CpGs is associated with aCA status. **Figure S5.** Shows the correlation between placental DNA methylation and gene expression at *IL6* locus. **Figure S6.** Shows no association of *IL6* expression with gestational age, fetal sex and preeclampsia status. (DOCX 1205 kb)

Additional file 2: **Table S1.** Table listing the allele counts and frequencies of the SNPs used in the statistical analyses. **Table S2.** Table listing the counts per genotype for IL6 SNPs based on the three clusters. (XLSX 20 kb)

## Abbreviations

450 k array: Illumina Infinium HumanMethylation450 BeadChip
850 k array: Illumina Infinium MethylationEPIC BeadChip
aCA: acute chorioamnionitis
AIM: Ancestry informative marker
CpG: Cytosine-phosphate-guanosine
eQTL: methylation quantitative trait loci
GA: Gestational age
GEO: Gene expression omnibus
HC: Hofbauer cells
IL6: Interleukin-6
KS: Kolmogorov-Smirnov
MDS: Multi-dimensional scaling
mQTL: methylation quantitative trait loci
ns: not significant
PE: Preeclampsia
PTB: Preterm birth
QC: Quality control
SNPs: Single nucleotide polymorphisms
sPTB: spontaneous preterm birth
TLR: Toll-like receptor
