# Supplementary material for: Association of a placental Interleukin-6 genetic variant (rs1800796) with DNA methylation, gene expression and risk of acute chorioamnionitis
Source: BMC Med Genet. 2019 Feb 22;20:36. doi: 10.1186/s12881-019-0768-0 (PMC6387541; doi:10.1186/s12881-019-0768-0)

**Figure S1. Association of SNP genotypes (rs1800795, rs1800796, and rs1554973) with ancestry in study cohort**. Ancestry is described as a continuous measure using the top three ancestry MDS coordinates. There were significant differences in the distribution of the top two ancestry MDS coordinates between the genotypes, however, ancestry MDS coordinate 3 was not significantly different between the genotypes for the three SNPs.

**
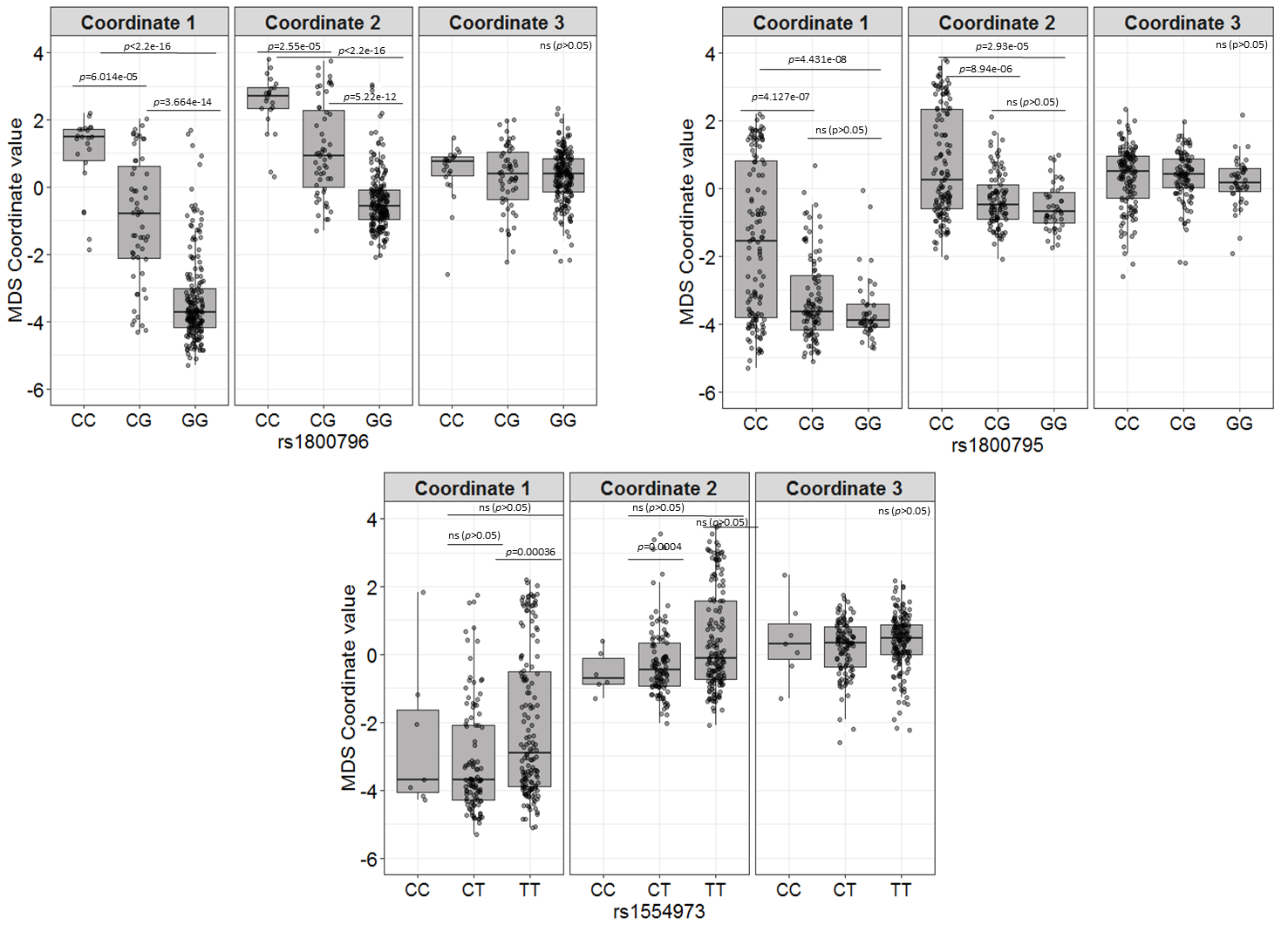
**

**Figure S2. Correlation of β values across eight *IL6*-related CpGs.** Using Spearman’s correlation, modest (r>0.5) to strong (r>0.7) correlations were observed across most of the *IL6*-related CpGs. Stronger correlations were observed among CpGs that were physically closer to one another (bps).


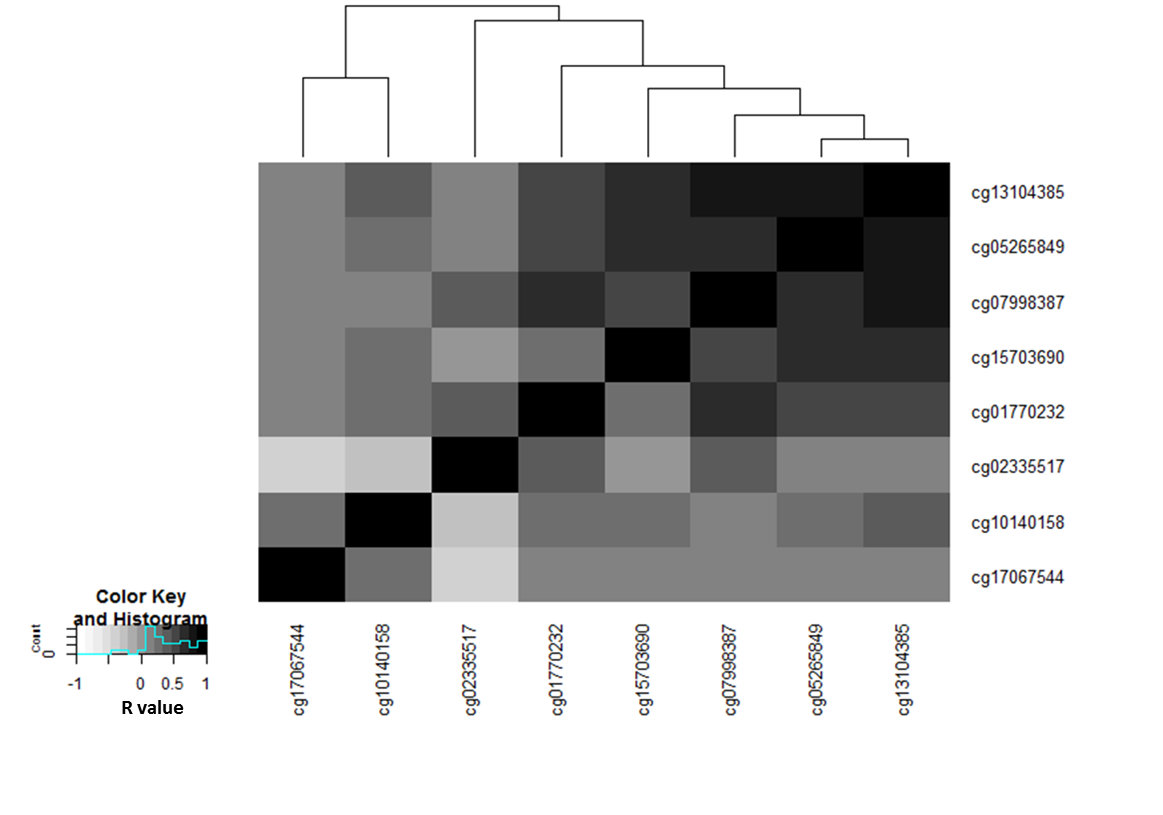


**Figure S3. Differential methylation of *IL6-*related CpGs based on *IL6* genotype status at rs1800796**. In a subset of the study population (n=67), individuals with CC genotype (n=9) showed increased DNAm levels compared to carriers of GG genotype (n=54) at five of the tested CpGs (Bonferroni-corrected p<0.05). As expected, the four heterozygotes (CG) showed intermediate DNAm levels.


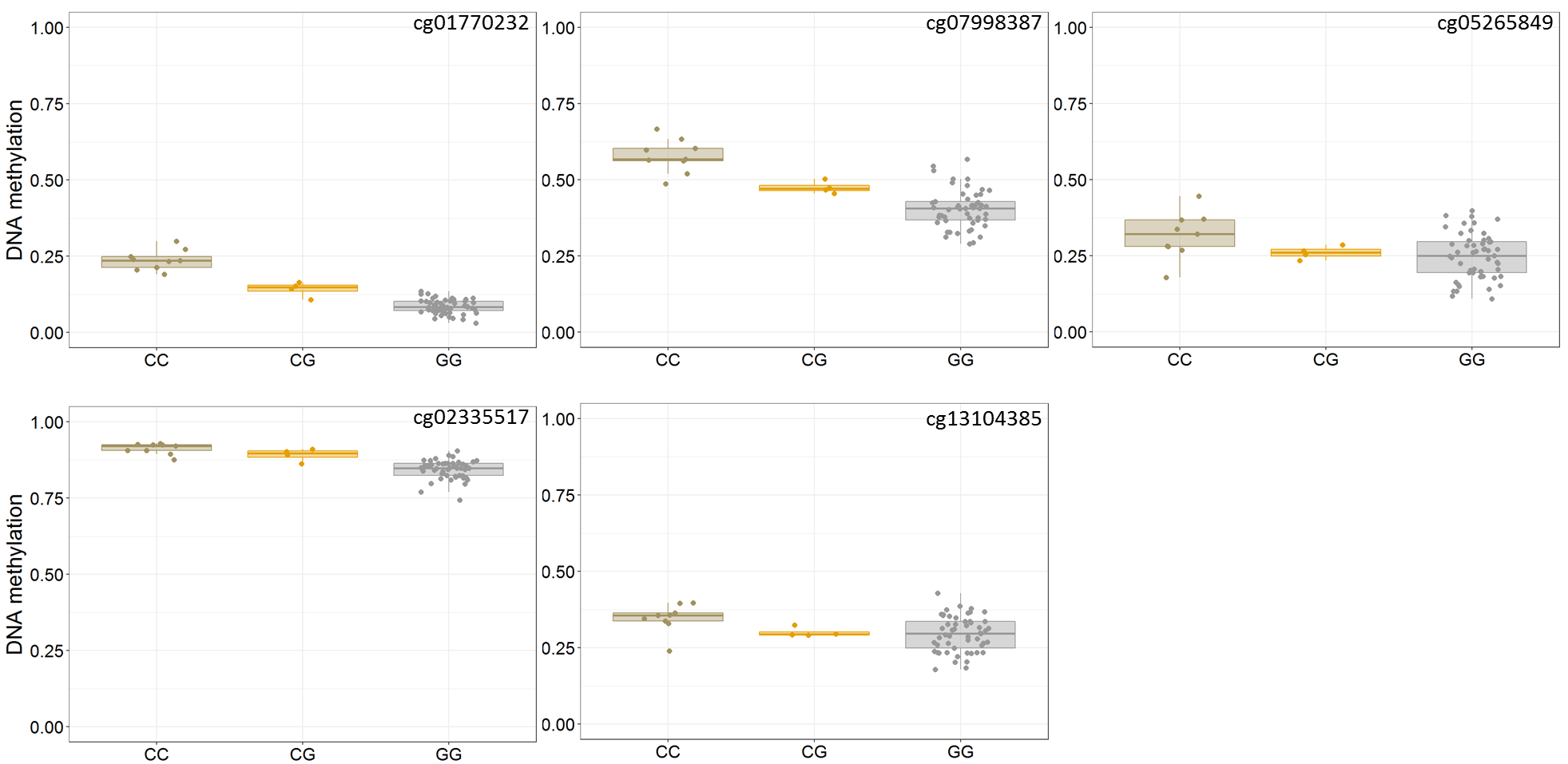


**Figure S4. Altered DNAm at *IL6-*related CpGs is associated with aCA status.** In a subset of the study population for which genotype and DNAm data was available (n=67), aCA-associated placentas showed increased DNAm compared to non-aCA associated placentas (*p*<0.05, Kruskal-Wallis test).


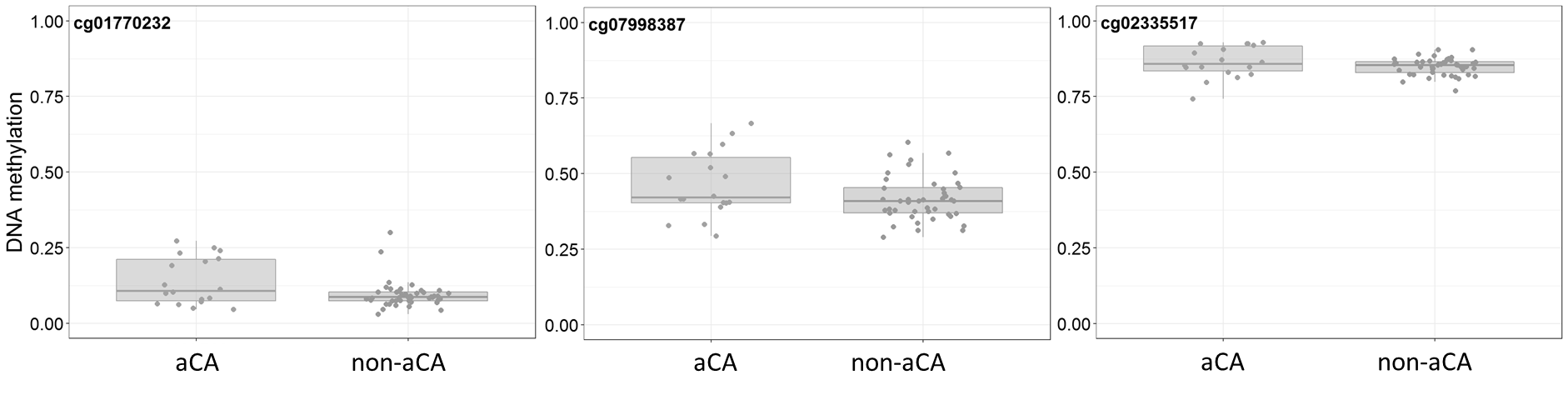


**Figure S5. Correlation between placental DNAm and gene expression at *IL6* locus**. A negative trend between DNAm and gene expression was observed in both the publically available datasets: 4a) GSE98224 (n=48 placentas); 4b) GSE44667 (n=16 placentas).


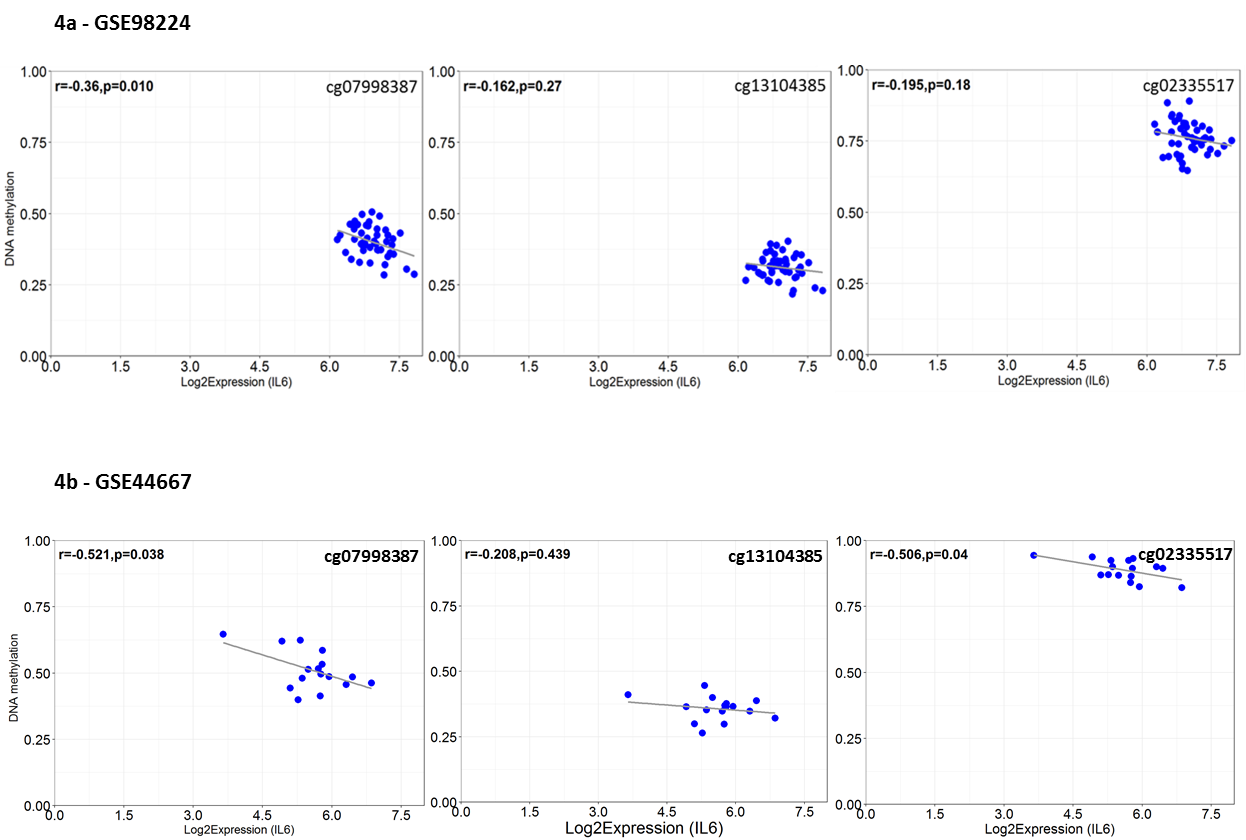


**Figure S6. No association of *IL6* expression with gestational age, fetal sex and preeclampsia status.** In each graph, *IL6* gene expression, measured as log2 transformed, is depicted on the y-axis in comparison to A) gestational age, B) fetal sex C) preeclampsia status


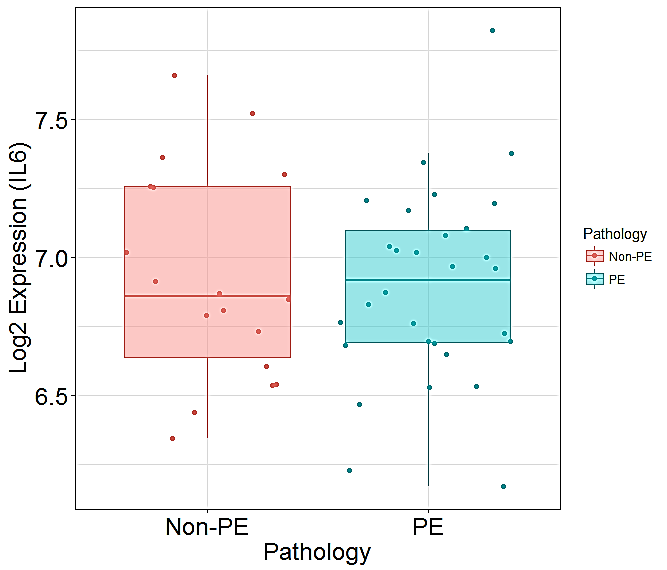

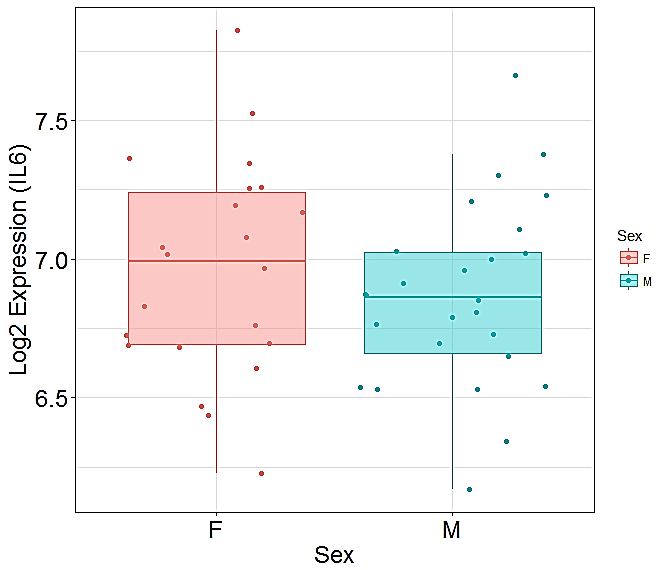


*p*-value = 0.75

*p*-value = 0.44

A

B

C


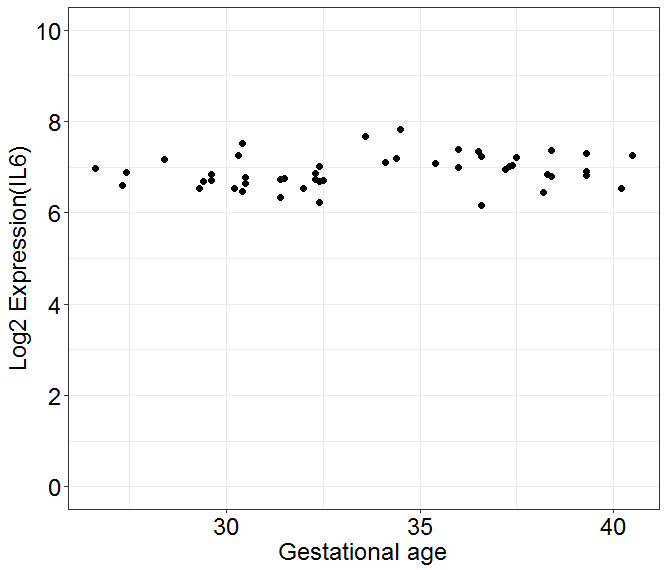

Supplement: Supplementary file 1 — Figure S1. Shows the association of SNP genotypes (rs1800795, rs1800796, and rs1554973) with ancestry in study cohort. Figure S2. Shows the correlation of β values across eight IL6-related CpGs. Figure S3. Shows differential methylation of IL6-related CpGs based on IL6 genotype status (rs1800796). Figure S4. Shows altered DNA methylation at IL6-related CpGs is associated with aCA status. Figure S5. Shows the correlation between placental DNA methylation and gene expression at IL6 locus. Figure S6. Shows no association of IL6 expression with gestational age, fetal sex and preeclampsia status. (DOCX 1205 kb) [file 12881_2019_768_MOESM1_ESM.docx]
